# Triiodothyronine acts on DAO to regulate pulmonary fibrosis progression by facilitating cell senescence through the p53/p21 signaling pathway

**DOI:** 10.3389/fphar.2024.1433186

**Published:** 2024-09-11

**Authors:** Xiaoshu Guo, Kai Xu, Lan Wang, Linke Ding, Wenwen Li, Xinsheng Zhang, Weiming Zhao, Ningdan Wang, Gaiping Wang, Wenyu Zhao, Ivan Rosas, Guoying Yu

**Affiliations:** ^1^ State Key Laboratory Cell Differentiation and Regulation, Henan International Joint Laboratory of Pulmonary Fibrosis, Henan Center for Outstanding Overseas Scientists of Organ Fibrosis, Institute of Biomedical Science, College of Life Science, Henan Normal University, Xinxiang, China; ^2^ Department of Physiology, Department of Fundamental Medicine, Changzhi Medical College, Changzhi, China; ^3^ Division of Pulmonary, Critical Care and Sleep Medicine, Baylor College of Medicine, Houston, TX, United States

**Keywords:** idiopathic pulmonary fibrosis, D-amino acid oxidase, triiodothyronine, anti-fibrotic axis, p53/p21 pathway

## Abstract

**Background:**

Idiopathic pulmonary fibrosis (IPF) is the result of multiple cycles of epithelial cell injury and fibroblast activation; currently, there is no clear etiology. Increasing evidence suggests that protein metabolism and amino acids play a crucial role in IPF, but the role of D-amino acids is not yet clear. The aim of this study was to identify novel mediators in order to test the hypothesis that D-amino acid oxidase (DAO) plays a significant role in the pathogenesis of IPF.

**Methods:**

We analyzed DAO gene expression in patients with IPF and mice with bleomycin (BLM)-induced lung fibrosis. We performed *in vitro* and *in vivo* assays to determine the effect of DAO on primary type II alveolar epithelial cells from mice and A549 cells.

**Results:**

DAO expression was downregulated in the lungs of IPF patients and BLM-induced fibrotic mice. Treatment with D-serine (D-Ser) or drug inhibition of DAO promoted cell senescence through the p53/p21 pathway. *Dao* ^−/−^ mice showed an intensified fibrotic response, and the anti-fibrotic role of T_3_ was abolished.

**Conclusion:**

We concluded that the DAO-p53/p21 axis might be a key anti-fibrotic pathway regulating the progress of fibrosis and facilitating the therapeutic role of T_3_.

## Introduction

Idiopathic pulmonary fibrosis (IPF) is a chronic progressive lung disease characterized by hyperplasia of alveolar epithelial cells, increased numbers of myofibroblasts, deposition of interstitial extracellular matrix, and remodeling of the lung structure (American Thoracic Society, 2000). Its clinical manifestations mainly include persistent cough and exertional dyspnea, which can be life threatening (Prêle et al., 2022; Wang et al., 2021). In recent years, the incidence of IPF has been continuously increasing, with a 5-year survival rate of 70%–80% after diagnosis (Hu et al., 2022). The currently known risk factors for IPF include age, smoking, gastroesophageal reflux, and environmental variables (American Thoracic Society, 2000; Xylourgidis et al., 2019; Ntatsoulis et al., 2021). However, in patients diagnosed with IPF, the course of this disease is progressive and irreversible, leading to severe pulmonary fibrosis and respiratory failure. Therefore, it is imperative to develop safe and effective treatment strategies for IPF.

Recent studies have shown that IPF is an epithelial cell-driven disease, and its pathogenesis involves processes such as DNA damage and cellular senescence (Spagnolo et al., 2021). Both endogenous and exogenous factors continuously induce DNA damage, triggering the DNA damage response (DDR) (Groelly et al., 2023). The DDR can maintain the genomic stability of cells through activating DNA repair mechanisms, cell cycle checkpoints, and apoptosis pathways. The activation of the DDR can lead to cellular senescence, which plays a significant role in the pathophysiological processes of IPF, particularly in relation to the progression of pulmonary fibrosis (Yao et al., 2023). Due to DNA damage, the p53 pathway is frequently activated, leading to cell cycle arrest in the G1 phase to allow sufficient time for damaged DNA repair. If DNA damage cannot be repaired, p53 can also induce cell apoptosis, preventing further growth and division of damaged cells to maintain genomic stability and inhibit tumor progression (Welz et al., 2022). In response to damage, the lung initiates a physiological fibrotic repair mechanism. If damage occurs repeatedly or if repair is excessive, it may lead to the development of pathological fibrosis. Pathological fibrosis can result in irreversible changes in the structure and function of damaged tissues, ultimately leading to organ dysfunction.

Through reanalysis of the NCBI public database (GSE47460), it was found that D-amino acid oxidase (DAO) is significantly downregulated in IPF patients, suggesting that DAO plays an important role in the pathogenesis of IPF. Flavin adenine dinucleotide is a coenzyme of DAO and is essential for its normal function. DAO is expressed in various organisms, including yeast and mammals (Chieffi et al., 2020). D-amino acids play important roles in biological processes, particularly in the modulation of neural signal transduction and host defense mechanisms. In neural signal transduction, D-amino acids may be involved in neurotransmitter synthesis and release, influencing communication between neurons. In host defense, D-amino acids may participate in immune responses and antimicrobial defense, contributing to the maintenance of microbial balance within the host and the prevention of pathogenic microbial infections (Yan et al., 2020). DAO is involved in regulating the concentration and metabolism of D-amino acids, mainly present in tissues such as the brain, kidneys, and liver. Its functions in the human body primarily include regulating neurotransmitter metabolism, influencing neural transmission, and modulating immune responses (Bastings et al., 2019; Sacchi et al., 2021). Previous research has found that DNA damage activates DAO, which can promote tumor cell senescence through the generation of reactive oxygen species clusters (Nagano et al., 2019). In addition, DNA damage typically triggers complex repair mechanisms and signaling pathways within cells, processes that are associated with cell senescence.

Previous studies have shown that in a bleomycin (BLM)-induced mouse model, triiodothyronine (T_3_) has a better anti-fibrotic effect than pirfenidone and nintedanib, which is related to its protective effect on the mitochondria of alveolar type II epithelial cells (AT2) (Yu et al., 2018). T_3_ may reduce fibrosis by affecting the expression and activity of matrix metalloproteinases, regulating the degradation of the extracellular matrix. In addition, T_3_ may also affect the number and activity of fibrosis-related cells by regulating pathways, such as cell proliferation and apoptosis, thereby influencing the degree of fibrosis. The systematic exploration of the impact of DAO on IPF in this study provides a more comprehensive theoretical basis for T_3_ as a potential therapeutic strategy for IPF.

## Materials and methods

### Human samples

The RNA sequencing data were downloaded from the publicly available Gene Expression Omnibus (GEO) database (GSE47460).

### Animal model

The CRISPR-AI system (Cyagen Biosciences Inc., Guangzhou, China, contract number: KOAI 190428JW1) was utilized to create DAO knockout C57BL/6 mice. Male and female mice aged 12–24 weeks, matched for age and sex, were included in this study. Each cage accommodated five mice, and they were maintained on a 12-h light/dark cycle. Animal studies and experimental procedures were approved by the Animal Ethics Committee of the College of Life Science, Henan Normal University (approval no. HNSD-2022BS0413). For the administration of BLM, the mice were anesthetized with isoflurane and subjected to bronchial instillation of 1.5 U/kg of BLM (Hanjiang Pharmaceutical) or an equivalent volume (50 μL) of 0.9% saline as the control. Between days 10 and 20 after BLM administration, the treatment group received aerosolized T_3_ (Sigma, T2877, USA; 40 μg/kg) every other day, and the mice were euthanized on the 21st day following an intraperitoneal injection of 20% Urethan (0.1 mL). Experimental protocols were approved by the Institutional Animal Care and Use Committee of Henan Normal University (approval No. HNSD-2022BS0413).

### Cell culture and treatment

A human lung adenocarcinoma A549 cell line (CL-0016, Procell) was cultured in DMEM/F12 medium (HyClone), and mouse primary type II alveolar epithelial cells (MIC-iCell-a005, Cellverse) were cultured in an iCell primary epithelial cell culture system (PriMed-iCell-001, Cellverse). The overexpression of DAO was achieved through transfection with pEGFP-C1-DAO (Wt) (WA6029Gn, Generay) or pEGFP-C1-Dao (Wt) (G0299353-1, Beyotime); siRNA-*DAO* (siG1457170458-1-5, Ribobio) was utilized to suppress the expression of *DAO*; 50 μM of 6-chloro-3-hydroxybenzisoxazole (CBIO, TS-02303, J&K) was employed to inhibit the enzyme activity of DAO. BLM (390302, Hanhui-pharma) and (D-Serine, D-Ser, 312-84-5, Acmec) were sourced from China.

### Cell scratch assay

A549 and iCell cells were seeded at a density of 5×10^6^ in a 6-well plate. Once the cells reached 90% confluence, cross scratches were created. Cells were then treated with D-Ser at final concentrations of 0, 0.2, 0.5, 2, 5, and 10 mM. Scratch healing was observed in A549 cells at 6 and 18 h, and in iCell cells at 24 and 48 h.

### CCK-8 assay

A549 and iCell cells were inoculated at a density of 3×10^3^ cells/100 µL/well into a 96-well plate with five replicate wells; no cell culture medium served as the blank control. Cells were allowed to adhere for 24 h and were then treated with D-Ser at final concentrations of 0, 0.2, 0.5, 2, 5, and 10 mM. A549 cells were treated for 48 h, while iCell cells were treated for 120 h. Then, 10 μL of CCK-8 solution was added per well in 100 μL of culture medium, and the cells were incubated in a CO_2_ incubator for 2 h. Finally, the absorbance was measured at 450 nm using a MULTISKAN GO microplate reader (China, Thermo Scientific).

### Transwell assay

A549 cells (10^5^) were inoculated into each well of a 24-well plate and cultured for 24 h. Cells were detached with serum-free medium, and the density was adjusted to 5×10^5^ cells/mL. The red fluorescent probe Dil (C1036, Beyotime) was added at a working concentration of 5 μM to stain the cells for 10 min. Then, 200 μL of the A549 cell suspension was placed in the upper chamber of a 12-well Transwell insert (3422, Corning), with 600 μL of complete culture medium containing 15% FBS in the lower chamber. D-Ser was added at a final concentration of 2 mM. PBS-treated wells served as controls. At 4 and 8 h, the number of A549 cells invading the lower chamber was observed under an inverted fluorescence microscope (Eclipse Ts2R, Nikon).

### SA-β-gal assay

A549 and iCell cells were transfected into a 24-well plate with 10^5^ cells per well using pEGFP-DAO (Wt) and pEGFP-Dao (Wt), followed by treatment with bortezomib and D-Ser. The cells were washed with PBS once, 250 μL of fixation solution was added per well, and the cells were fixed at room temperature for 15 min. Routine PBS washes (3 min × 3) were performed. Then, 250 μL of reaction mixture was added per well (2.5 μL A solution +2.5 μL B solution +232.5 μL C solution + X-Gal). The plates were incubated overnight at 37°C (non-CO_2_ incubator). The staining solution was then removed, and 2 mL of PBS was added, followed by imaging with an inverted fluorescence microscope (Eclipse Ts2R, Nikon).

### Histology, Western blot, immunohistochemistry, and immunofluorescence

The mouse lung tissues were fixed in 4% paraformaldehyde, embedded in paraffin, and sectioned into 4 μm slices. The sections were dewaxed to water before use, and then photographed using a Nikon E200 microscope (Nikon, Tokyo, Japan) or a pathology slide scanner (3DHISTECH, China and Hungary). The image viewing software programs were K-Viewer and SlideVewer. Hematoxylin and eosin (HE) staining was performed using a kit (C0105S, Beyotime) with the following steps: stain with hematoxylin for 7 min; rinse in tap water to remove excess stain for approximately 10 min; rinse in distilled water for 5 s; stain with eosin for 1 min; dehydrate, clear, and mount. Masson’s trichrome staining was performed (G1340, Solarbio) via the following steps: fix the tissues in Bouin’s solution (prepared by mixing 1.22% picric acid saturated solution, 37% formaldehyde solution, and glacial acetic acid in a volume ratio of 15:5:1) overnight at 4°C; wash the tissues in 70%–80% ethanol; rinse with running water until the yellow color disappears; stain with Weigert’s iron hematoxylin for 7 min; rinse slightly with running water, and place in dH_2_O; apply a bluing reagent (as needed); counterstain with acid fuchsin for 5 min; wash with 1% glacial acetic acid for 1 min; wash with 1% phosphomolybdic acid for 5 min (control under the microscope until collagen fibers turn purple-blue); stain with aniline blue for 1–5 min (observe under the microscope); wash with 1% glacial acetic acid for 1 min; dehydrate quickly in 95% ethanol 5 times; rapidly dehydrate in absolute ethanol for 2 s; clear in xylene until optimal transparency (observe under the microscope); mount with neutral resin.

The Western blot procedure was as follows: 10% SDS-PAGE vertical slab protein gel electrophoresis; constant current of 100 mA for 4 h wet transfer onto PVDF membrane; membrane washing with TBST, 5 min × 3; blocking with 5% skim milk at room temperature for 1 h; incubation at 4°C overnight with rabbit polyclonal antibodies against DAO (1:1000), N-Cadherin (1 μg/mL), α-SMA (1 μg/mL), p53 (1:1000), p-p53 (1:1000), p21 (1:1000), β-actin (1:5000), and GAPDH (1:10000); membrane washing with TBST, 5 min × 3; incubation with goat anti-rabbit secondary antibody (1:2000 to 1:5000) at room temperature for 1.5 h; membrane washing with TBST; visualization using ECL.

The steps of immunohistochemistry were as follows: antigen heat retrieval in citrate buffer at 100°C for 10 min; PBS wash, 5 min; endogenous peroxidase blocking at room temperature for 10 min; PBS wash, 5 min × 3; incubation with 0.3% Triton X-100 for 10 min; PBS wash, 5 min × 3; immunohistochemistry serum blocking at room temperature for 30 min; α-SMA/THR alpha/THR beta rabbit polyclonal antibody 1:100, overnight incubation at 4°C; PBS wash, 5 min × 3; goat anti-rabbit secondary antibody, 1:100, 37°C incubation for 30 min; PBS wash, 5 min × 3; observation under a light microscope until satisfactory DAB staining, then terminate staining; counterstain with hematoxylin; routine dehydration, clearing, and mounting.

The steps of immunofluorescence were as follows: double distilled water wash for 5 min; incubation with 0.3% Triton X-100 at room temperature for 10 min; PBS wash, 5 min × 3; blocking with 30% serum at room temperature for 30 min; incubation overnight at 4°C with a mixture of DAO mouse monoclonal antibody at 1:100 and SP-C rabbit polyclonal antibody at 1:100, followed by PBS wash, 5 min × 3; incubation at 37°C with goat anti-rabbit and goat anti-mouse fluorescent secondary antibodies at 1:200 for 30 min, followed by PBS wash; imaging after DAPI nuclear staining and mounting.

The primary antibodies were purchased from Affinity USA (DAO, DF7266; β-actin, AF7018; GAPDH, AF7021; THR alpha, AF9218; THR beta, DF6477; SP-C, DF6647), Abcam USA (α-SMA, ab5694; N-Cadherin, ab18203; Goat Anti-Rabbit IgG H&L, ab6721), CST USA (p53, CST2527; p-p53, CST9287; p21, CST2947), Santa Cruz USA (DAO, sc-398757), and Beyotime (Alexa Fluor 647-labeled Goat Anti-Rabbit IgG, A0468; Alexa Fluor 488-labeled Goat Anti-Mouse IgG, A0428).

### Quantitative PCR

The concentration of each transcript was normalized to the β-actin mRNA level using the 2^−ΔΔCT^ method. Trizol (79306, QIAGEN), a miRNeasy Mini kit (217004, QIAGEN), a GoScript Reverse Transcription System (A5001, Promega), and a QuantiNova SYBR Green PCR kit (208052, QIAGEN) were used in accordance with the manufacturer’s protocols. Primers from Sangon were utilized (Table 1).

**TABLE 1:** Information on gene primers.

Genes		Primer sequence (5′to 3′)	Annealing temperature (°C)
*Dao*	F	GGT​TCC​AAG​ACA​GTT​ACA​CTC​G	58
	R	AGG​GTG​GGC​TCC​AGT​TTA​CA	
*DAO*	F	CCCCAACAACCCACAGGA	58
	R	GCC​CGA​GAT​TAG​GAA​CAG​G	

*Dao* refers to the primers for gene amplification in mouse tissue; *DAO*, denotes the primers used for amplification in human cell line A549.

### Lung hydroxyproline, serum T_3_, FT_3_, and rT_3_ levels

The hydroxyproline assay kit (MAK008) was purchased from SIGMA. The procedure was as follows: Homogenize 10 mg of lung tissue in 100 μL of ddH_2_O; add 100 μL of concentrated hydrochloric acid to 100 μL of tissue homogenate and heat at 120°C for 3 h; centrifuge at room temperature, 10000 g × 3 min; take 10 μL of the supernatant and place it in a 96-well plate; set up 6 standard wells, each with 10 μL, with concentrations of 0, 0.2, 0.4, 0.6, and 0.8 µg/well; add 6 µL of Chloramine T concentrate and 94 µL of Oxidation Buffer to each well, and incubate at room temperature for 5 min; add 50 µL of DMAB concentrate and 50 µL of Perchloric Acid to each well, and heat at 60°C for 90 min; measure absorbance at 560 nm using a MULTISKAN GO microplate reader (China, Thermo Scientific).

The serum levels of T_3_, FT_3_, and rT_3_ obtained from the mice were detected using ELISA kits (JL13028, JL20674, JL143442, J&L Biological, China), following the manufacturer’s protocols. First, 50 μL of sample serum was added to each well. Biotin-labeled antibody (50 μL; standard, except for the blank well) was added to each well and incubated at 37°C for 30 min. The liquid was discarded and the plate was tapped gently, followed by washing with 1× washing solution for 1 min (repeated 5 times); horseradish peroxidase-labeled antibody (100 μL) was added (except for the blank well), followed by incubation in a 37°C water bath for 30 min; the liquid was discarded and the plate was tapped gently, followed by washing with 1× washing solution for 1 min (repeated 5 times); 50 μL of chromogenic agents A and B was added and incubated at 37°C for 15 min; the OD value of each well was measured with 50 µL of stop solution at a wavelength of 450 nm (within 15 min).

### Molecular docking

The molecular docking study using Autodock Vina 1.2.2 (http://autodock.scripps.edu/) for model visualization involved obtaining the molecular structure of compound T_3_ from the PubChem compound database (https://pubchem.ncbi.nlm.nih.gov/). The 3D coordinates of the protein DAO (PDB code, 7U9U; resolution, 1.66 Å) were downloaded from the PDB (http://www.rcsb.org/). The protein and ligand files were prepared by converting all protein and molecule files to PDBQT format, removing all water molecules, and adding polar hydrogen atoms. The grid box was centered to cover the structural domain of each protein and adapt to free molecular motion. The docking pocket was set as a square pocket of 30 Å × 30 Å × 30 Å, with a grid spacing of 0.05 nm.

### Statistical analysis

The data are presented as mean ± SEM. Group differences were compared using a non-paired Tukey multiple comparison test or one-way analysis of variance. All tests were two-tailed, and *p* ≤ 0.05 was considered significant. Data analysis was performed using Prism 7.0 software (GraphPad).

## Results

### DAO expression was downregulated in the lungs of IPF patients and BLM mouse models

**FIGURE 1:**
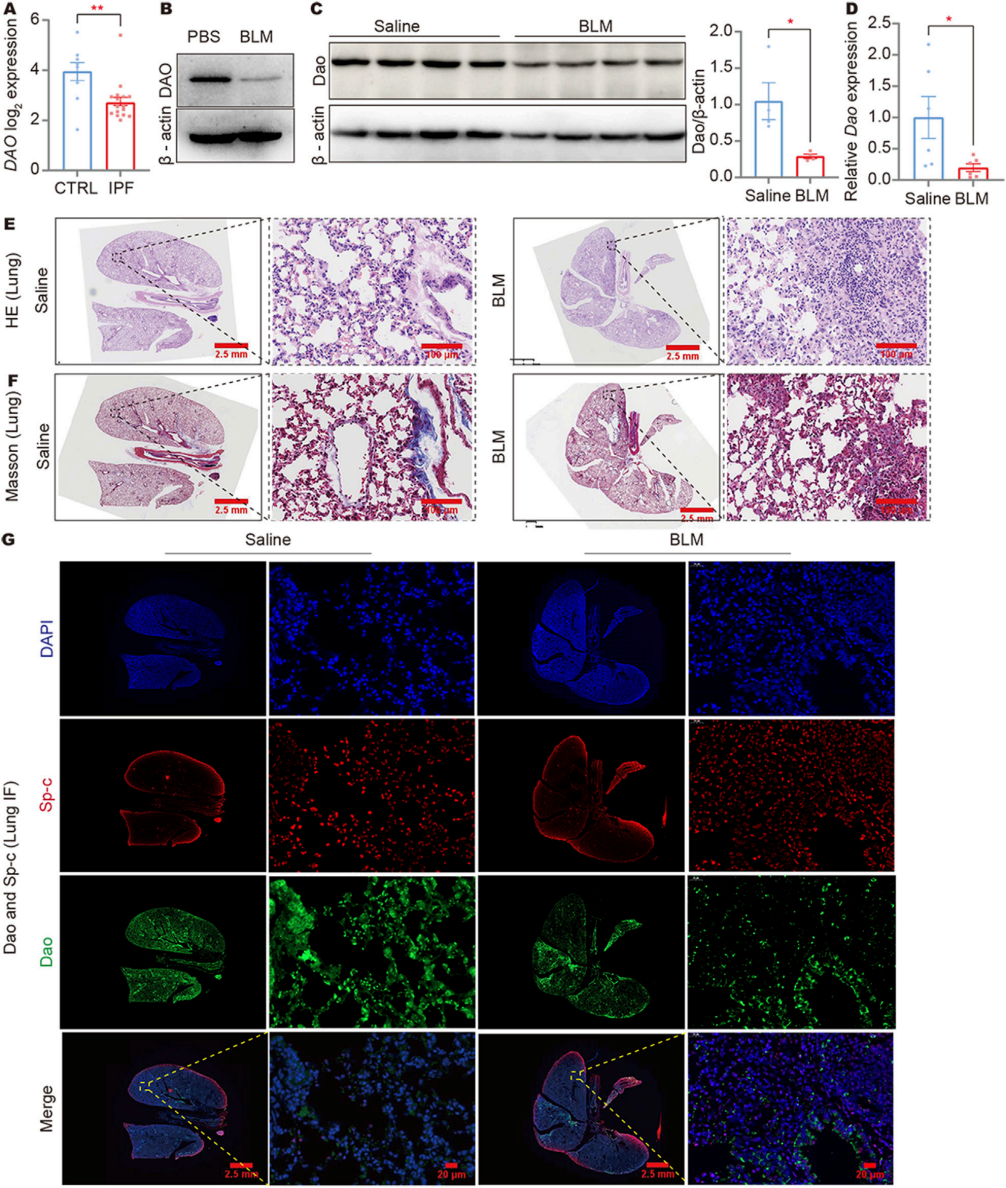
DAO expression was downregulated in IPF lungs and BLM-induced fibrotic mouse lungs. **(A)** The results of IPF gene sequencing showed that DAO expression was significantly lower in IPF patients than in the controls; **(B)** A549 cells treated with 0.02 U/mL of BLM for 24 h. Representative immunoblot image of the whole-cell lysate of the DAO protein expression; **(C)** Representative immunoblot images (up) and densitometry analysis (down) of the *Dao* protein expression; **(D)** RT-qPCR analysis of the *Dao* expression levels in mouse lungs; **(E)** Representative images of HE staining of mouse lung paraffin sections (Saline control, left; BLM treated, right); **(F)** Representative images of Masson’s staining of mouse lung paraffin sections (Saline control, left; BLM treated, right); **(G)** Representative *Dao* and Sp-c immunofluorescence staining images of mouse lung paraffin sections showing that *Dao* expression was downregulated and Sp-c expression was upregulated. *p < 0.05, **p < 0.01.

In a reanalysis of the NCBI public database (GSE47460), it was discovered that the expression of the DAO gene was significantly decreased in the lungs of IPF patients (Figure 1A). To establish an idiopathic fibrosis model, BLM-challenged A549 cells and C57BL/6N mice were used. The examination of BLM-treated A549 cells revealed a reduction in DAO expression (Figure 1B). Furthermore, both immunoblot (Figure 1C) and real-time quantitative PCR analysis (Figure 1D) demonstrated decreased Dao expression in the fibrotic mouse lungs challenged with BLM. The HE and Masson’s staining results showed that the alveolar structure disappeared in the mouse lung tissue after BLM treatment, with increased blue collagen fibers and pathological changes in lung consolidation (Figures 1E, F). Immunofluorescence double labeling of mouse lung tissue showed an increase in Sp-c-positive cells in the lung consolidation area after BLM treatment, but a decrease in Dao-positive expression (Figure 1G).

### *Dao* knockout promoted BLM-induced pulmonary fibrosis in mice

**FIGURE 2:**
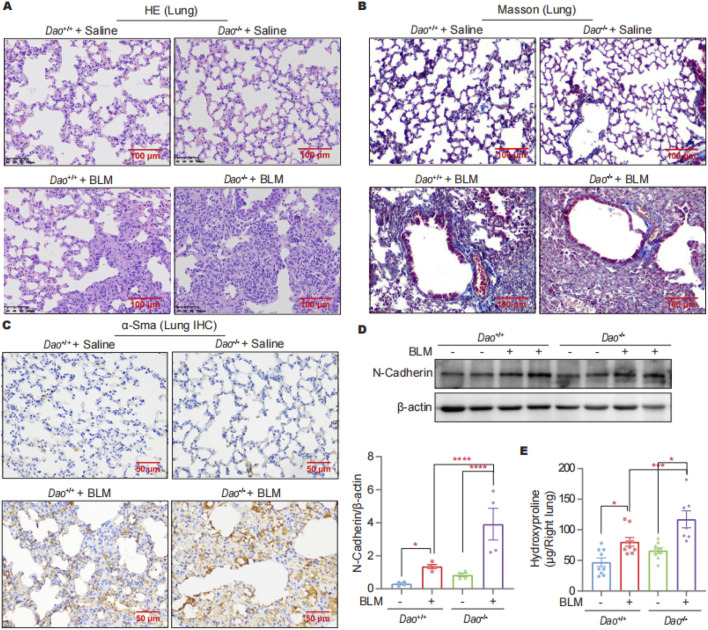
*Dao* knockout promoted the BLM-induced pulmonary fibrosis. **(A)** Representative HE staining images of mice challenged with BLM or saline; **(B)** Representative Masson’s staining images of mice challenged with BLM or saline; **(C)** Representative immunohistochemistry staining images of α-Sma in lung samples; **(D)** Representative immunoblot images of N-Cadherin in mouse lung lysates; **(E)** Hydroxyproline content of the indicated groups. *p < 0.05, ***p < 0.001, ****p < 0.0001.

The HE staining results showed no significant differences between *Dao*-wild-type (*Dao* ^*+*/+^) + Saline and *Dao*-knockout (*Dao* ^*−/−*^) + Saline groups for lung tissue morphology, whereas the lung tissue of *Dao* ^−/−^ + BLM group exhibited more pronounced fibrosis and pulmonary consolidation compared to the *Dao* ^+/+^+BLM group (Figure 2A). Compared to *Dao* ^*+*/+^ mice, *Dao* ^*−/−*^ mice exhibited increased collagen deposition in the lungs following the BLM challenge, as demonstrated by Masson’s staining (Figure 2B). Additionally, there was a significant increase in the expression of the myofibroblast markers alpha-smooth muscle actin (α-Sma)^*−/−*^ (Figure 2C) and N-Cadherin (Figure 2D) in the lungs of *Dao* ^*−/−*^ mice, along with a notable elevation in hydroxyproline content (Figure 2E). These findings indicate that *Dao* ^*−/−*^ mice displayed heightened susceptibility to BLM-induced pulmonary fibrosis.

### D-ser suppresses the proliferation and migration of A549 cells

**FIGURE 3:**
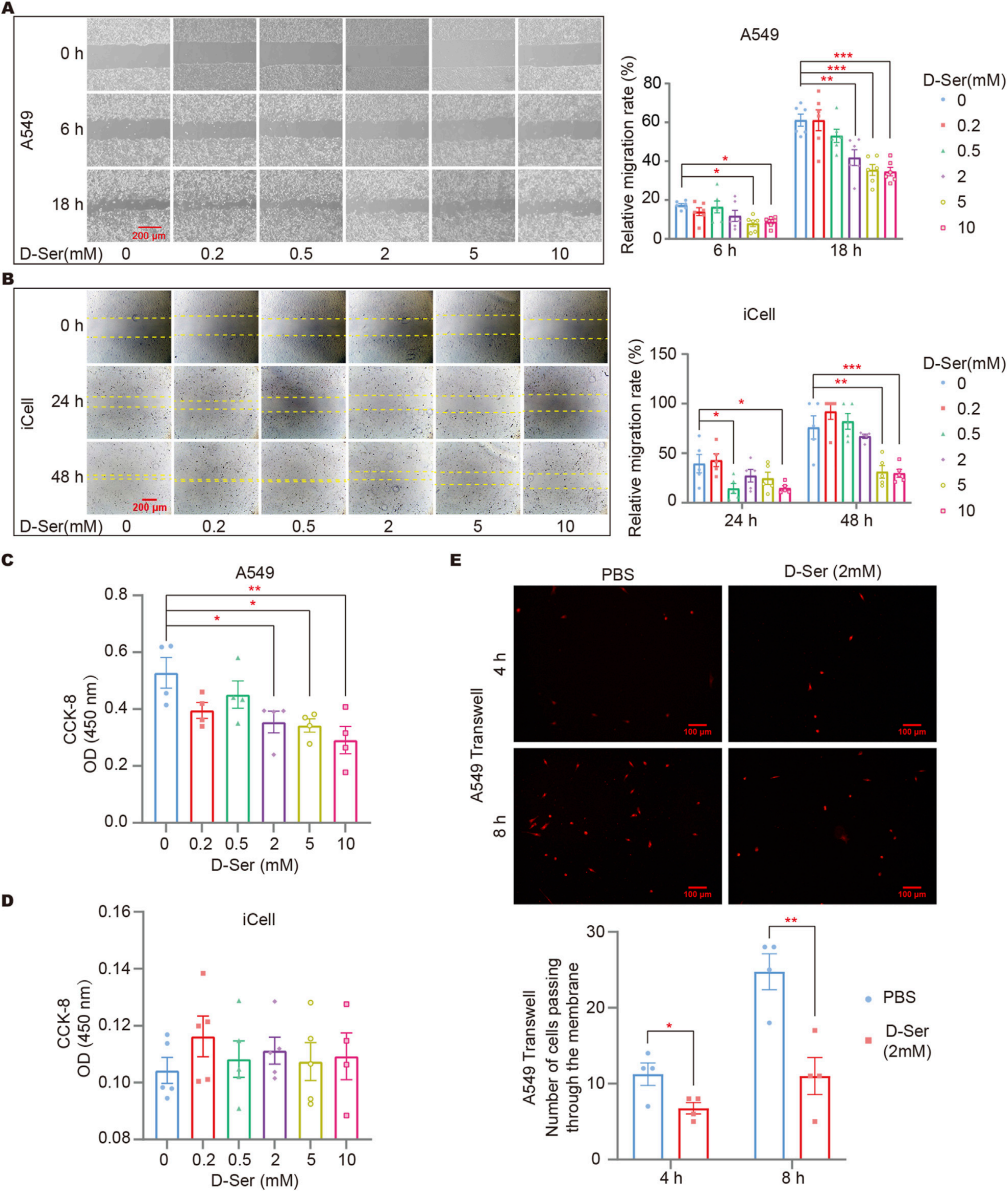
D-Ser inhibited migration of A549 cells and iCell cells. The migration rate of A549 **(A)** and iCell **(B)** cells was determined by a cell scratch assay under various concentrations of D-Ser. The cell viability of A549 **(C)** and iCell **(D)** cells was determined by CCK-8. **(E)** A549 cells were treated with 2 mM of D-Ser, and cell migration was determined by a transwell assay. *p < 0.05, **p < 0.01, ***p < 0.001.

A cell scratch assay was conducted to assess cell migration in response to D-Ser. Gradient concentrations of 0, 0.2, 0.5, 2, 5, and 10 mM of D-Ser were applied to observe the wound healing process of A549 and iCell cells. We found that 0.5 mM D-Ser inhibits the migration of A549 and iCell cells, while higher concentrations of 5 mM and 10 mM D-Ser significantly inhibit the migration (Figures 3A, B). CCK-8 was used to examine cell viability in response to various D-Ser concentrations. The results showed that 2 mM or higher concentrations of D-Ser significantly inhibited the proliferation of A549 cells, while iCell cells showed little to no response up to 10 mM. D-Ser had a minor impact on the proliferation of iCell cells (Figures 3C, D). Additionally, 2 mM of D-Ser significantly inhibited the migration of A549 cells in a transwell assay (Figure 3E).

### D-ser is a direct factor in the induced senescence of A549 cells

**FIGURE 4:**
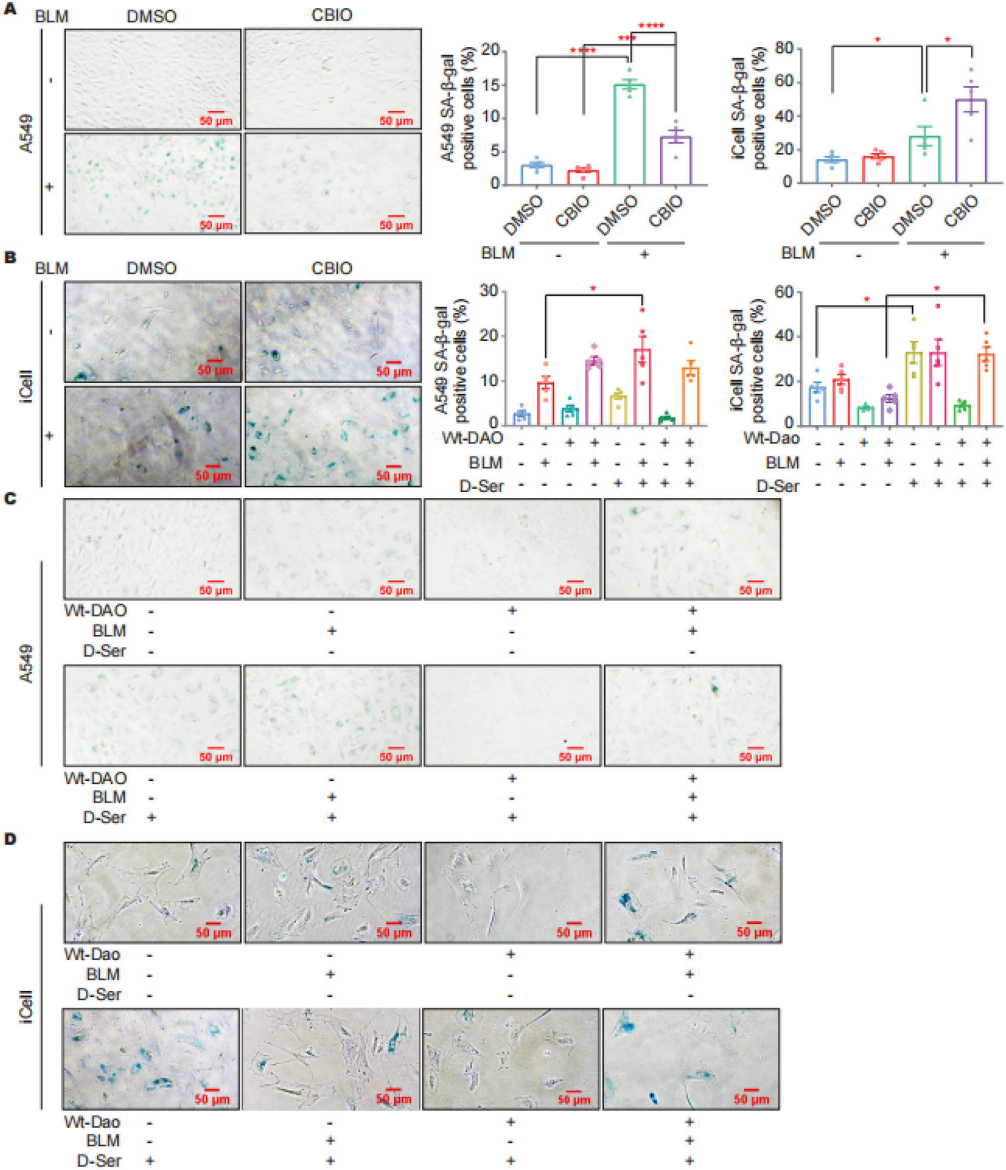
DAO affected the senescence of A549 and iCell cells. **(A)** A549 cells were treated with 50 μM of CBIO for 24 h; then, 0.01 U of BLM was added for an additional 24 h, and then SA-β-gal staining was performed; **(B)** iCell cells (procedure was consistent with **(A)**); **(C)** A549 cells were transiently transfected with pEGFP-DAO for 24 h; then, 0.01 U of BLM and 2 mM of D-Ser were added. Following 72 h, the cells were stained with β-galactosidase; **(D)** iCell cells (procedure was consistent with **(C)**). *p < 0.05, ***p < 0.001, ****p < 0.0001.

SA-β-gal staining was performed on cells with DAO overexpression and after inhibiting DAO enzyme activity in response to BLM treatment (0.01 U/mL, a sublethal dose of BLM). The staining results indicated that CBIO treatment can reduce BLM-induced cellular senescence in A549 cells (Figure 4A). In contrast, inhibition of *Dao* enzyme activity in iCell cells by CBIO increased BLM-induced cellular senescence (Figure 4B). D-Ser significantly increased BLM-induced senescence in A549 cells. Interestingly, transfection with DAO did not reduce senescence in A549 cells but rather increased it (Figure 4C). The combination of DAO enzyme activity inhibition and reduced senescence in A549 cells suggests characteristics typical of tumor cells, where increased DAO expression and activity can inhibit the proliferation of malignant type II alveolar epithelial cells. In iCell cells, D-Ser significantly increased senescence, while transfection with Wt-Dao significantly reduced BLM-induced senescence (Figure 4D).

### DAO regulated BLM-induced cell senescence through p53/p21

**FIGURE 5:**
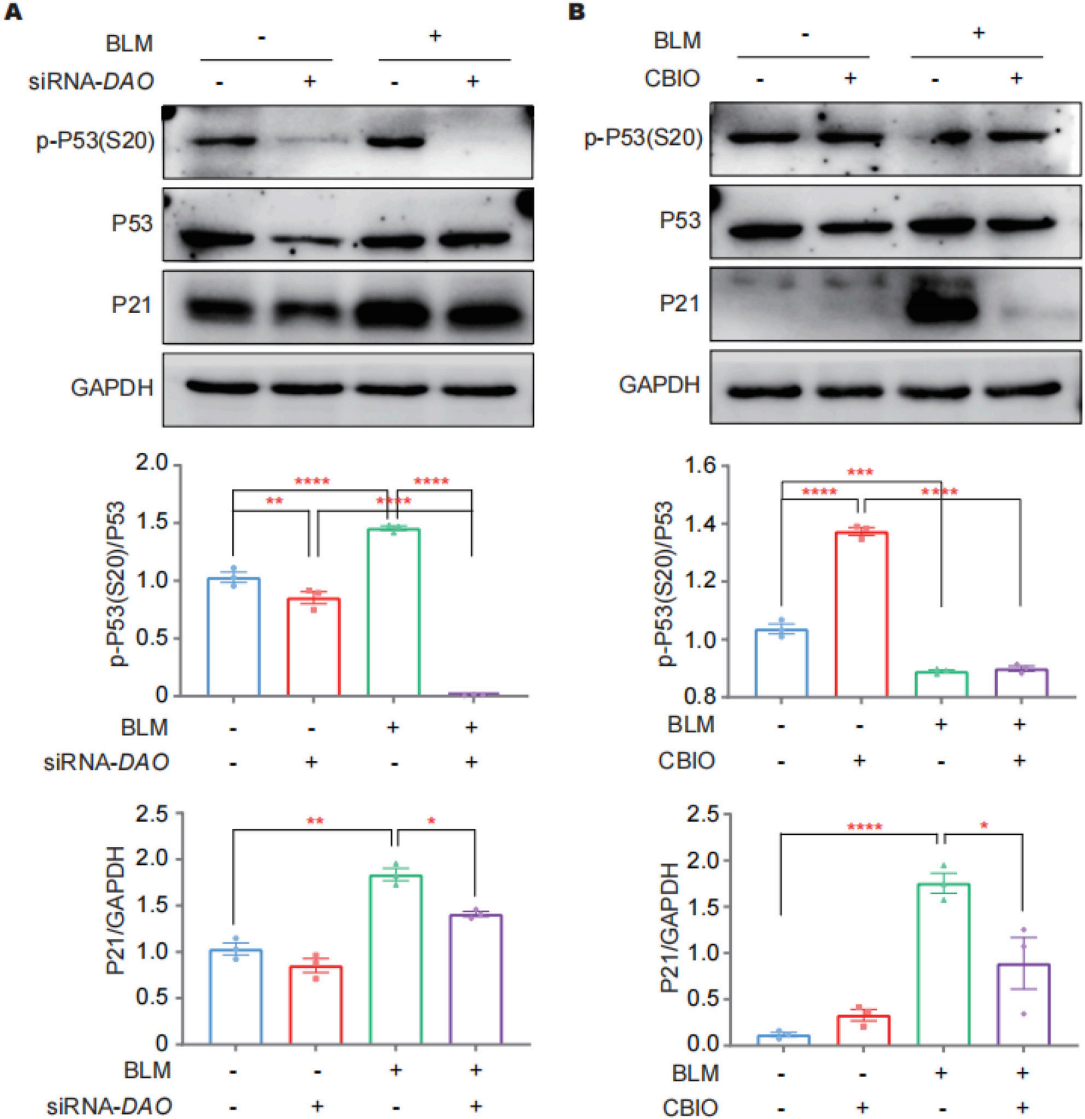
DAO regulates A549 cell senescence through p53/p21. **(A)** A549 cells were transfected with siRNA-DAO for 24 h; then, 0.02 U of BLM was added for an additional 24 h, and immunoblotting was performed to detect the expression levels of P21, P53, and p-P53 (S20) in the cell lysates; **(B)** A549 cells were treated with 50 μM of CBIO for 24 h; subsequently, 0.02 U of BLM was added for an additional 24 h, and immunoblotting was performed, as in **(A)**. *p < 0.05, **p < 0.01, ***p < 0.001, ****p < 0.0001.

Immunoblotting was performed to investigate the abundance of total P21, P53, and phosphorylated P53 at Ser20 [p-P53 (S20)] in A549 cells as the key players in the DDR following *DAO* knockdown and enzyme activity inhibition. P53 plays a critical role in the cell cycle, with cell cycle arrest in senescent cells largely relying on increased p-P53 (S20). P21, a cyclin-dependent kinase inhibitor downstream of p-P53 (S20), inhibits CDK1 and prevents the cell cycle from progressing into the G1/S phase. The results revealed that the administration of BLM led to a significant increase in the expression of P21 and p-P53 (S20) in A549 cells. Knockdown of DAO resulted in a significant decrease in the BLM-induced expression of P21, P53, and p-P53 (S20) (Figure 5A). Furthermore, pharmaceutical inhibition of enzyme activity by CBIO significantly reduced the upregulation of P21 induced by BLM (Figure 5B). These findings were consistent with the SA-β-gal staining rsults, further suggesting that reduced DAO expression or inhibition of DAO enzyme activity may enhance the proliferation of type II alveolar epithelial cells and contribute to malignant transformation.

### The interaction between T_3_ and DAO and their role in pulmonary fibrosis

**FIGURE 6:**
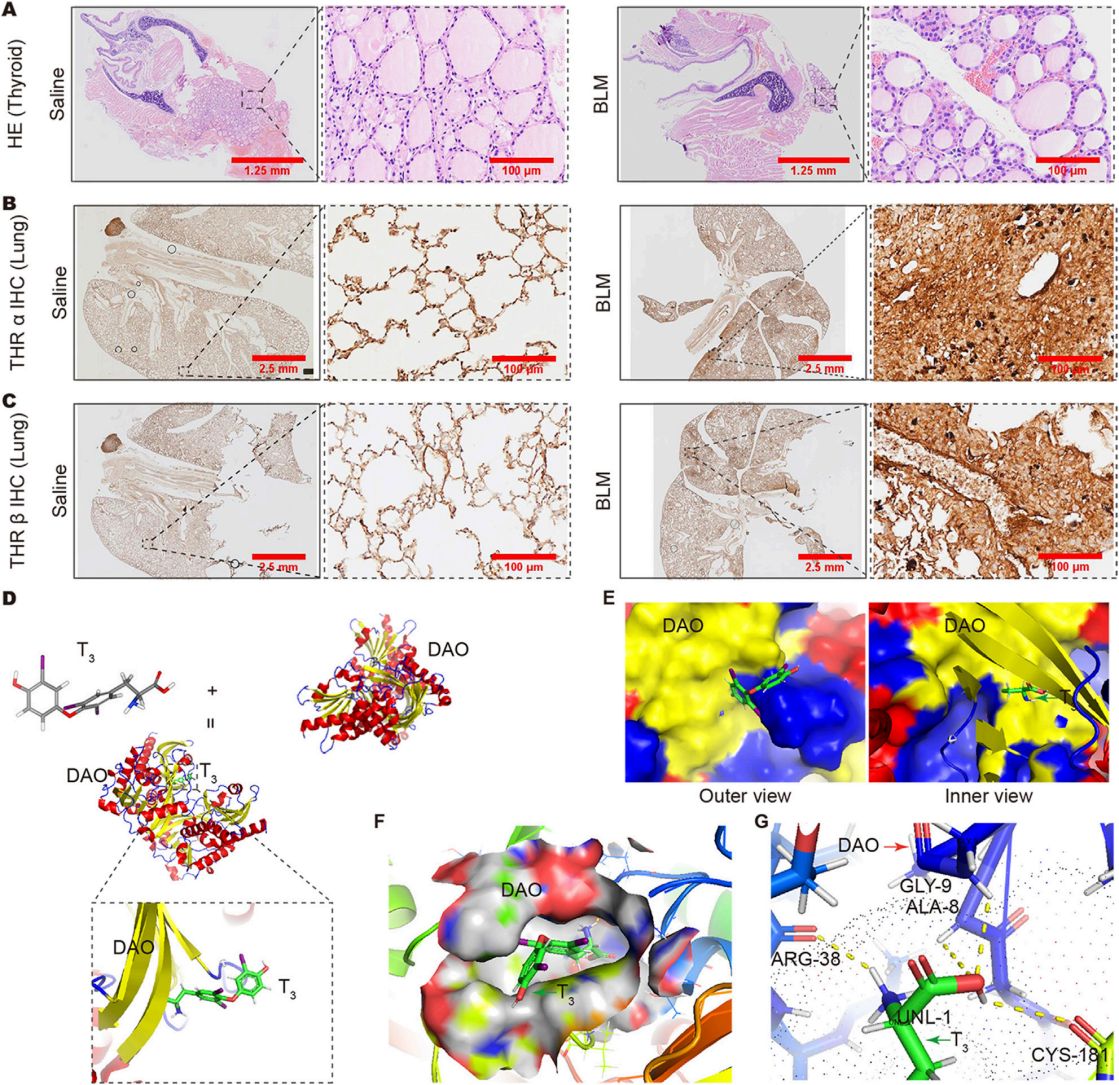
Changes in thyroid and pulmonary thyroid hormone receptors and the analysis of the binding capacity between T_3_ and DAO. **(A)** The HE staining results showing full thyroid follicles in the BLM group mice, and the follicular cells exhibit a proliferative morphology compared to the Saline group; **(B)** The immunohistochemistry results showing increased expression of THRα receptors in the lungs of BLM-treated mice compared to the Saline group; **(C)** The immunohistochemistry results showing an increased expression of THRβ receptors in the lungs of BLM-treated mice compared to the Saline group; **(D)** T_3_ molecular formula, DAO protein, cartoon diagram of DAO protein binding with T_3_, and a magnified view of the binding site; **(E)** DAO protein and T_3_ binding surface type local magnification diagram (protein outer view and protein inner view); **(F)** PyMOL software displayed the 3D structure of the DAO binding pocket. The coloring ranges from magenta (for strong H-bonds) to green (for weak H-bonds). The color gradient goes from magenta (for strong hydrophobic regions) to blue (for less hydrophobic regions); **(G)** The binding between the amino acid residues at the interface of T_3_ and DAO is formed by hydrogen bonds.

In the HE staining results, the thyroid follicles of mice treated with BLM were full, and the follicular cells showed a proliferative state (Figure 6A). Immunohistochemistry results showed that the expression of thyroid hormone receptors THRα and THRβ in the fibrotic areas of the lung increased in mice with lung fibrosis after BLM treatment (Figures 6B, C). All these results indicate a deficiency in thyroid hormones. To evaluate the affinity between T_3_ and DAO, we conducted molecular docking analysis. The binding positions of T_3_ and DAO proteins were obtained using Autodock Vina v.1.2.2. Figure 6D presents the 3D animation and magnified local binding site of the DAO protein after docking with T_3_, while Figure 6E shows the surface-type magnified outer and inner views of the DAO protein after docking with T_3_. The PyMOL software displayed the 3D structure of the DAO binding pocket. The coloring ranges from magenta (for strong H-bonds) to green (for weak H-bonds) and from magenta (for strong hydrophobic regions) to blue (for less hydrophobic regions) (Figure 6F). The top ten binding energies for the interaction between T_3_ and DAO were −7.412, −7.229, −6.829, −6.771, −6.724, −6.621, −6.587, −6.552, and −6.468 kcal/mol, indicating highly stable binding. DAO interacts with T_3_ through amino acid residues such as CYS-181, ARG-38, GLY-9, and ALA-8 to form hydrogen bond interactions (Figure 6G).

### The anti-fibrotic effect of T_3_ is associated with dao expression

**FIGURE 7:**
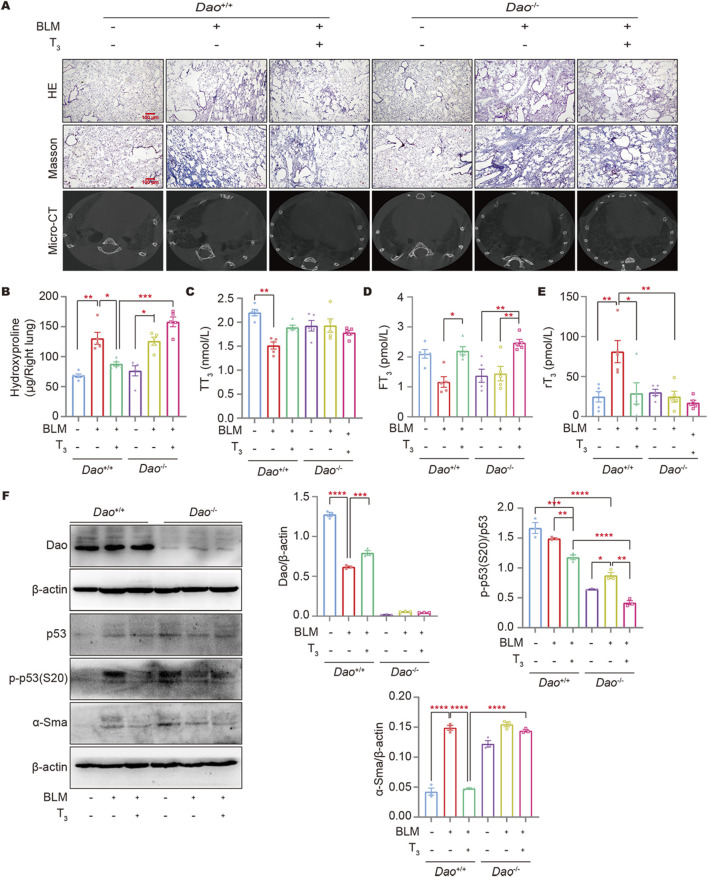
Dao is essential for the anti-fibrotic role of T_3_. **(A)** Representative images of mouse lungs after HE staining, Masson’s staining, and micro-CT; **(B)** The hydroxyproline content in mouse lung (right) homogenate; **(C–E)**: The levels of TT_3_, FT_3_, and rT_3_ in the peripheral blood of mice from each treatment group were determined by ELISA; **(F)** The levels of Dao, α-Sma, p53, and p-p53 (S20) were determined by Western blotting. *p < 0.05, **p < 0.01, ***p < 0.001, ****p < 0.0001.

HE, Masson’s staining, and micro-CT results showed that T_3_ treatment significantly improved the lung architecture of the BLM-challenged mice and reduced collagen deposition (Figure 7A). Consistently, T_3_ treatment notably decreased the augmented hydroxyproline content in the BLM-challenged wild-type mice. However, no therapeutic effect of the T_3_ treatment was observed in *Dao* ^*−/−*^ mice (Figure 7B). Affected individuals have low serum T_3_ but elevated rT_3_, an inactive form of T_3_, and inappropriately normal TSH levels, recapitulating the non-thyroidal illness syndrome in IPF subjects. Following the BLM challenge, serum T_3_ was significantly decreased (Figure 7C), and rT_3_ was significantly increased in wild-type mice (Figure 7E). The aerosolized delivery of T_3_ significantly increased the serum FT_3_ in mice (Figure 7D), reducing rT_3_ with a minimal effect on the total serum T_3_ levels. Western blot results showed that T_3_ treatment increased *Dao* expression in wild-type mice caused by BLM injury and significantly downregulated the levels of α-SMA, and p-p53 (S20). In *Dao* ^*−/−*^ mice, the therapeutic effect of T_3_ against BLM-induced pulmonary fibrosis was diminished (Figure 7F).

## Discussion

DAO activity may be involved in regulating the pathophysiology of humans through D-amino acid metabolism (Kim et al., 2019). This study explored the role of DAO in the pathogenesis of IPF. The results showed that the expression levels of DAO were significantly decreased in fibrotic lung tissues of IPF patients and BLM-induced mice, as well as in BLM-treated A549 cells. Through *Dao* and Sp-c fluorescent double labeling experiments, we found that in mice with BLM-induced pulmonary fibrosis, the area of type II alveolar epithelial cells increased, while *Dao* expression decreased. Type II alveolar epithelial cells are considered to be the driving factors of lung fibrosis (Parimon et al., 2020). These results suggest that DAO may be involved in the pathogenesis of pulmonary fibrosis. Moreover, treatment with D-Ser or pharmacological inhibition of DAO can promote cell senescence through the p53/p21 pathway. Lastly, *Dao* ^−/−^ mice showed enhanced lung fibrotic response, and the anti-fibrotic effect of T_3_ disappeared, suggesting that the anti-fibrotic effect of T_3_ depends on the expression levels of DAO.

We found that the expression level of DAO in pulmonary fibrotic tissue was significantly decreased. In addition, *Dao* ^−/−^ mice were more susceptible to BLM-induced pulmonary fibrosis compared to *Dao* ^+/+^ mice, confirming the correlation between DAO expression and the process of pulmonary fibrosis. The reduced expression of DAO may lead to the accumulation of D-amino acids in lung tissue, thereby affecting neural signal transduction and host defense mechanisms. Furthermore, DAO plays an important role in regulating amino acid metabolism and immune responses in tissues (Oldham et al., 2022). Therefore, the decreased expression of DAO in pulmonary fibrotic tissue may impact the metabolism and immune function of lung tissue, exacerbating the development of IPF.

*In vitro* experiments have shown that D-Ser at concentrations of 2 mM and above can significantly inhibit the proliferation and wound healing of A549 cells. In scratch assays, high concentrations of D-Ser also inhibited the migration of mouse primary type II alveolar epithelial cells (iCell cells). To avoid cell differentiation, the iCell cells were cultured in 2% FBS, resulting in slow proliferation. We continuously observed the cells for 5 days until they filled the 96-well plate, and the results of the CCK-8 assay did not show significant differences. The A549 cells used in our study were tumor cells, which influenced our interpretation of the results. Results from *in vitro* and *in vivo* studies found that BLM-induced DNA damage leads to decreased levels of DAO in aging mice and A549 cells, further exacerbating cell senescence. Cell senescence is characterized by sustained cell cycle arrest, accumulation of DNA damage, and excessive production of ROS, leading to the secretion of various inflammatory factors and age-related diseases (Matsuda et al., 2020). Single-cell sequencing results have shown that BLM can induce cell cycle arrest in mouse lung fibrosis AT2 cells (Strunz et al., 2020; Müller et al., 2021). This study used BLM to simulate DNA damage in A549 cells to induce fibrosis. In response to genotoxic stress, the DNA activates p53 and induces its downstream protein p21 (a biomarker of cell senescence) (Kumari and Jat, 2021). ROS, a byproduct of D-Ser degradation that inhibits DAO, is a product of various types of oxidative stress damage. In addition to activating p53, ROS accumulate widely in the aging process induced by various external factors. There is a positive feedback loop between ROS production and DNA damage, leading to cell senescence through p21 (Zhang et al., 2023).

This study used CBIO and specific siRNA fragments to respectively inhibit the enzyme activity and expression of DAO in A549 cells. We observed that inhibiting enzyme activity or downregulating the expression of DAO can alleviate BLM-induced senescence. We also observed that transfection with Wt-DAO in A549 cells exacerbated BLM-induced senescence. At this point, the data from *in vitro* and *in vivo* are inconsistent. The overexpression of DAO alone does not induce cell senescence, but it promotes BLM-induced cell senescence when combined with BLM, suggesting that DNA damage is the reason for DAO activation. This may be related to the malignant behavior of A549 cells. In addition, D-Ser promotes cell aging based on BLM damage, suggesting that D-Ser may be a substrate for DAO in DNA damage-induced senescence. These results also suggest that upregulating DAO expression plays a role in inhibiting the malignant transformation of type II alveolar epithelial cells. We confirmed in primary mouse iCell cells that CBIO inhibition of *Dao* activity can increase BLM-induced senescence, while transfection of Wt-*Dao* to overexpress *Dao* can alleviate BLM-induced senescence.

A prior study demonstrated that the thyroid hormone (T_3_) is a promising drug candidate for pulmonary fibrosis. The administration of aerosolized T_3_ reduced the severity of BLM-induced lung fibrosis in mice without affecting serum T_3_ levels. BLM caused a reduction in lung compliance, which was reversed by treatment with aerosolized T_3_, significantly enhancing the survival rate of mice exposed to a lethal dose of BLM (3.0 U/kg) (Yu et al., 2018). We first observed the effect of BLM on the thyroid of mice, which showed a proliferative response rather than pathological changes in mice with pulmonary fibrosis. This, along with the increased thyroid hormone receptor in the fibrotic zone of the lungs, confirms the potential impact of thyroid hormone deficiency on this disease. We further used computer simulation techniques to simulate the pocket structure of DAO and the binding mode of T_3_, both confirming the possibility of T_3_ influencing lung fibrosis progression through DAO.

Furthermore, after the BLM challenge, we observed that *Dao* ^−/−^ mice exhibited exacerbated lung fibrosis. *Dao* ^−/−^ mice did not display a typical response to T_3_ secretion and did not manifest the low T_3_ syndrome observed in *Dao* ^+/+^ mice following the BLM challenge. Upon T_3_ treatment, both *Dao* ^−/−^ mice and *Dao* ^+/+^ mice demonstrated elevated serum FT_3_ levels, indicating the adequacy of our therapeutic dosage. The decrease in serum rT_3_ levels in wild-type mice post-BLM challenge suggested that T_3_ administration could lower the levels of inactive T_3_ in the bloodstream. These findings indicate that DAO plays a vital role in fibrosis development and enhances the therapeutic effects of T_3_.

Although the tissue pathological changes induced by BLM in mice with pulmonary fibrosis are similar to those seen in humans, animal models have limitations, including a self-healing tendency in fibrosis and the inability of the lung epithelium to reshape under IPF (O’Dwyer and Moore, 2018; Zhang et al., 2020; Redente et al., 2021). In addition, primary AT2 cells cultured *in vitro* are prone to differentiate into AT1-like cells and are difficult to maintain. Therefore, we used the A549 cell line for *in vitro* experiments; however, A549 cells possess tumor cell characteristics and may not fully reflect normal lung cell properties (Nasonovs et al., 2021; Obraztsova et al., 2020).

## Conclusion

This study found that the expression levels of DAO were significantly decreased in IPF and BLM-induced mouse fibrotic lung tissues. Additionally, D-Ser treatment or pharmacological inhibition of DAO could promote cellular senescence through the p53/p21 pathway. Finally, the anti-fibrotic effect of T_3_ depends on the expression levels of DAO. Thus, DAO may play an important role in the process of pulmonary fibrosis and enhance the therapeutic effect of T_3_.

## Data Availability

The original contributions presented in the study are included in the article/Supplementary Material, further inquiries can be directed to the corresponding author.
