# Supplementary material for: Triiodothyronine acts on DAO to regulate pulmonary fibrosis progression by facilitating cell senescence through the p53/p21 signaling pathway
Source: Front Pharmacol. 2024 Sep 11;15:1433186. doi: 10.3389/fphar.2024.1433186 (PMC11422212; doi:10.3389/fphar.2024.1433186)
Supplement: Supplementary file 1 [file Presentation1.pptx]

## Slide 1
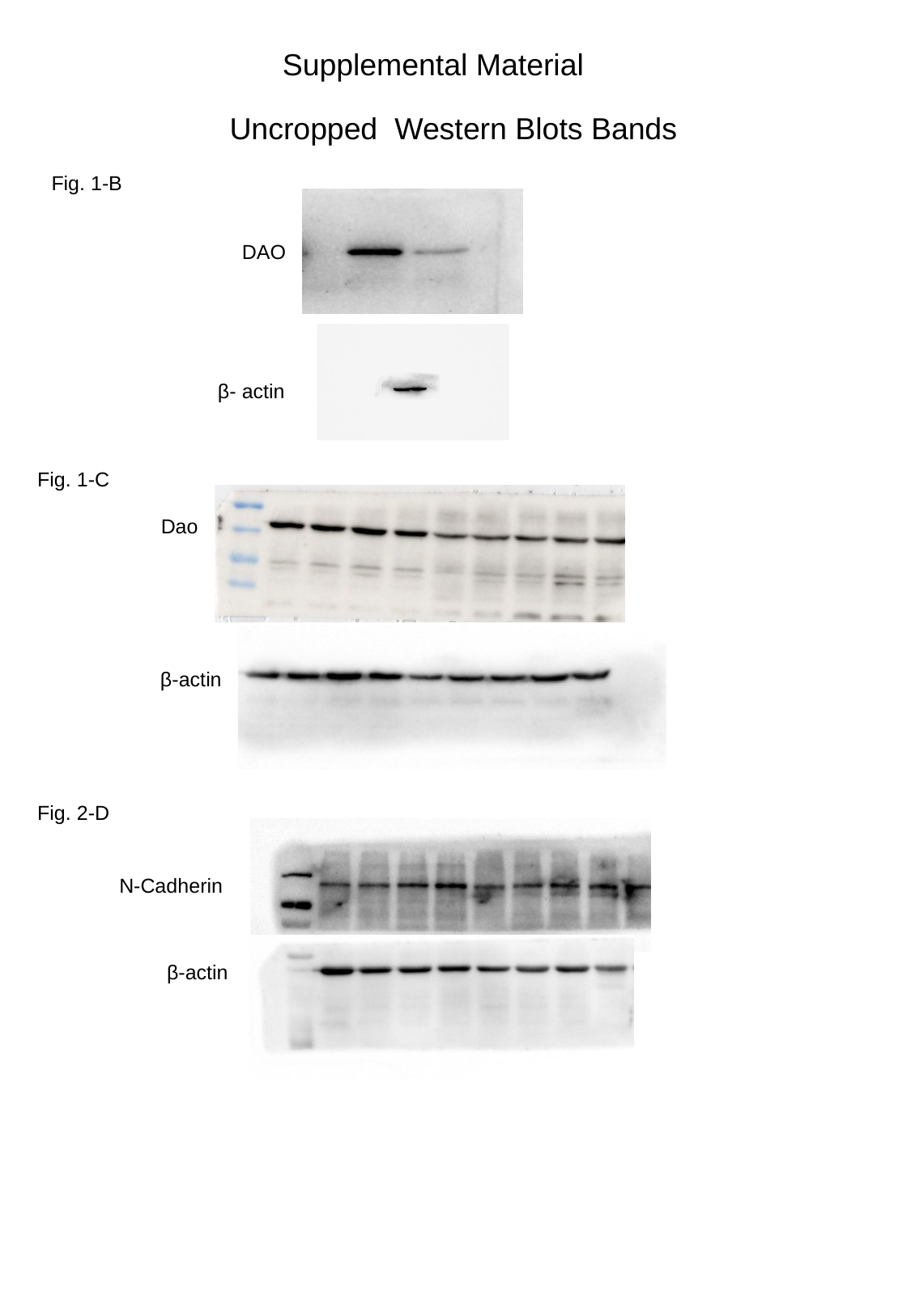

Supplemental Material
Uncropped Western Blots Bands
Fig. 1-B
DAO
β- actin
Fig. 1-C
Dao
β-actin
Fig. 2-D
N-Cadherin
β-actin

## Slide 2
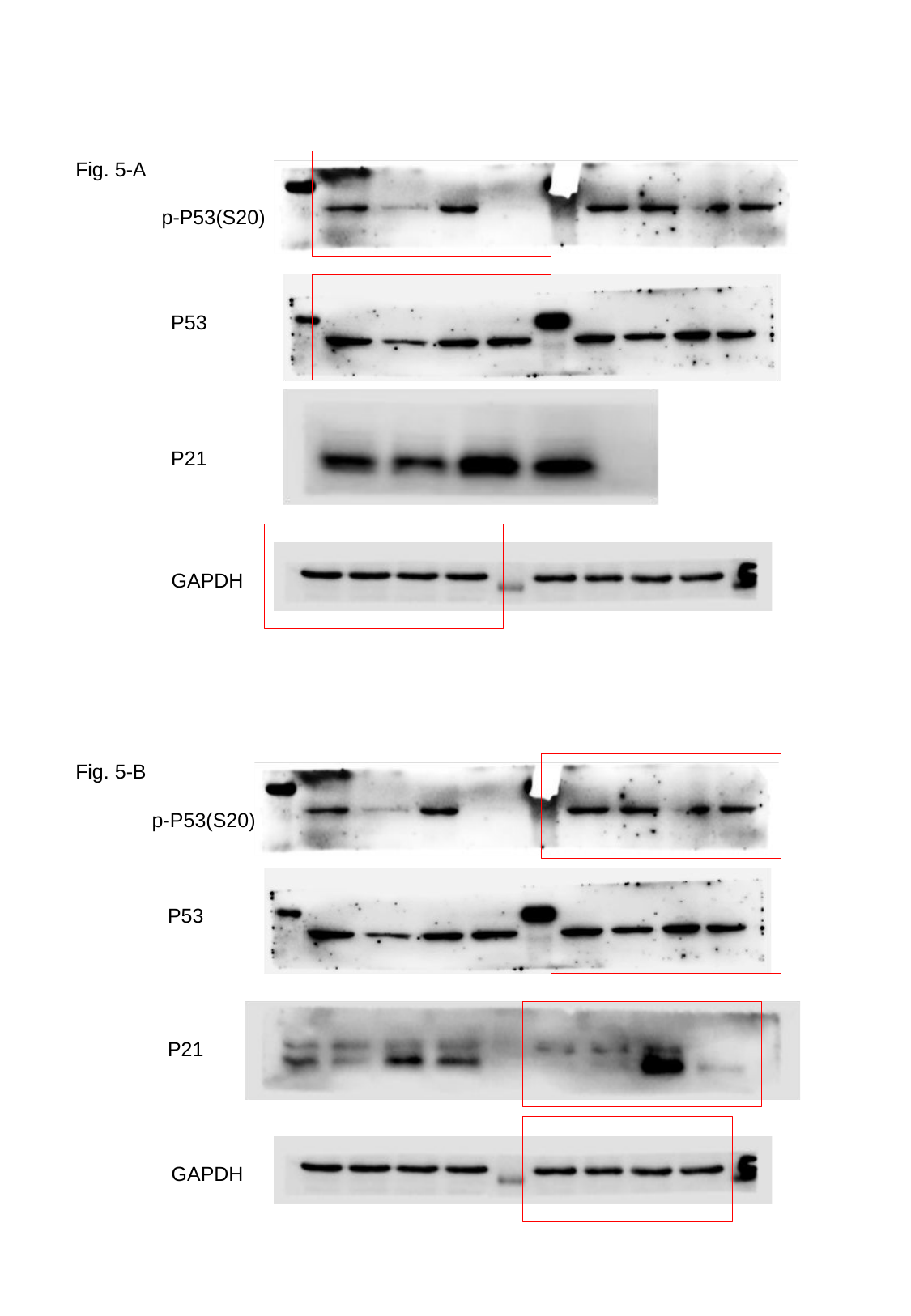

Fig. 5-A
p-P53(S20)
P53
P21
GAPDH
Fig. 5-B
p-P53(S20)
P53
P21
GAPDH

## Slide 3
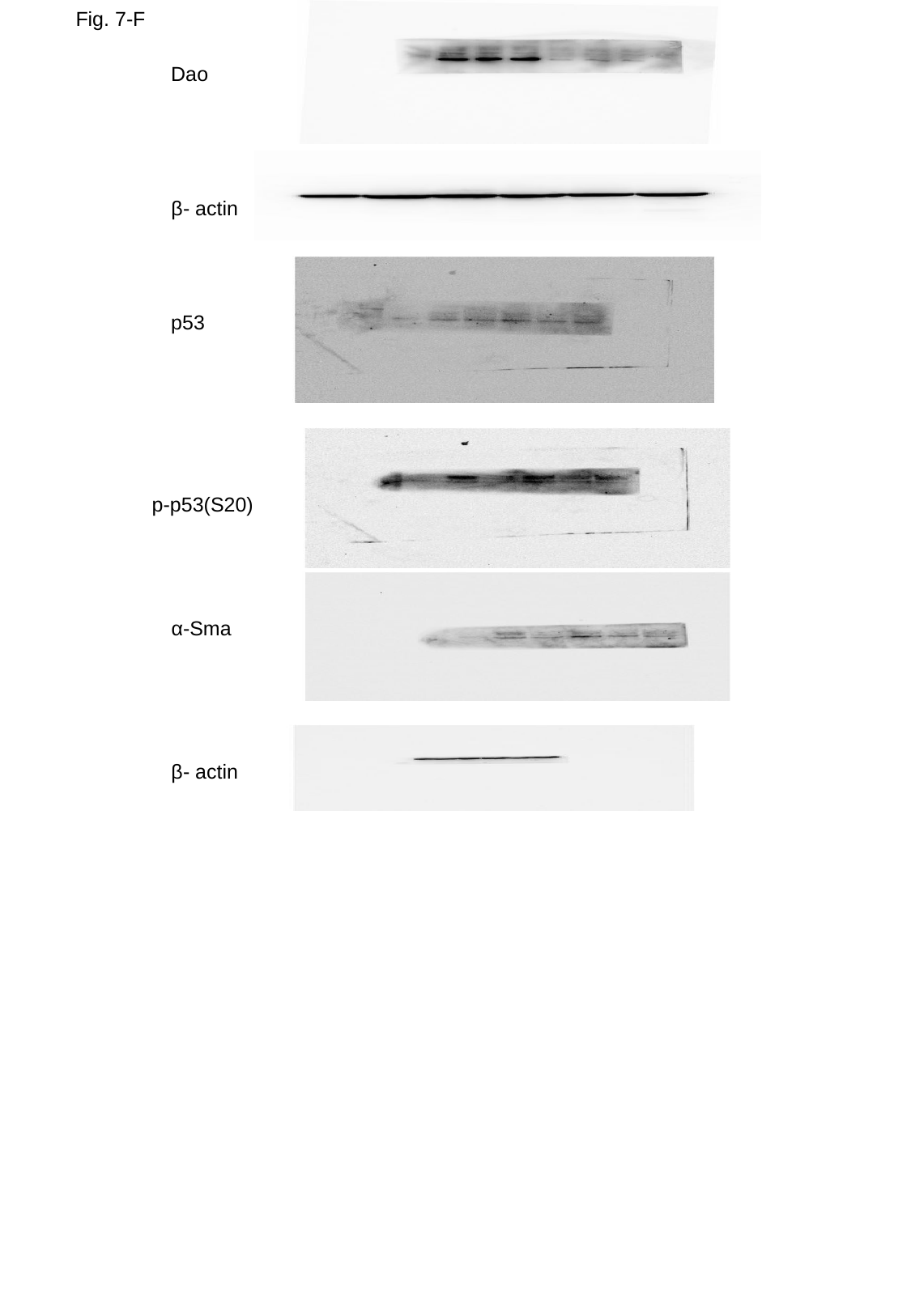

Fig. 7-F
Dao
β- actin
p53
p-p53(S20)
α-Sma
β- actin
